# Atypical Social Rank Recognition in Autism Spectrum Disorder

**DOI:** 10.1038/s41598-019-52211-8

**Published:** 2019-10-30

**Authors:** Shino Ogawa, Mayuko Iriguchi, Young-A Lee, Sakiko Yoshikawa, Yukiori Goto

**Affiliations:** 1 Kokoro Research Center, Kyoto University, Kyoto, 606-8501 Japan; 2 Department of Neurobiology and Behavior, Nagasaki University, Nagasaki, 852-8521 Japan; 3 Department of Food Sciences and Nutrition, Daegu Catholic University, Gyeongsan, Gyeongbuk 38430 South Korea; 4 Primate Research Institute, Kyoto University, Inuyama, Aichi 484-8506 Japan

**Keywords:** Social behaviour, Human behaviour, Autism spectrum disorders

## Abstract

Social animals, including humans, structure social groups where social hierarchy exists. Recognizing social rank of other group members is a crucial ability to subsist in such environments. Here we show preliminary evidence with a relatively small number of samples that children with autism spectrum disorder, a neurodevelopmental disorder involving social dysfunction, exhibit atypical, and more robust recognition of social rank than normal children, which may be developed to compensate deficits of the neural systems processing social information.

## Introduction

Autism spectrum disorder (ASD) is a neurodevelopmental disorder, whose prevalence has substantially been increasing recently, yet the reason remains unknown^1^. A primary symptom of ASD is social communication deficits, which could be associated with deficits of social cognition, such as Theory of Mind^2^. Accordingly, for instance, ASD subjects exhibit difficulties in assessments of emotions and thoughts of others from social signals such as facial expressions and behavioral intentions^3^.

Social animals, including humans, that spend their lives in social groups construct social hierarchy within the groups. Since social hierarchy is a principal determinant for allocation of limited resources for individuals in groups^4^, precise assessments of social rank of individuals in the groups is a crucial ability to live and behave advantageously in such social environments. Given that judging social rank of others is often based on social contexts (social signs, such as body languages, gestures and facial features, provoking social rank)^5,6^, which could also be associated with assessments emotional states and thoughts of others that are thought to be disrupted in ASD^3^, it is possible that ASD may also involve impairments of social rank recognition, which in turn, result in social communication deficits and maladaptation to the society. However, whether social rank recognition is altered in ASD has mostly remained unclear.

Adults, children, and even infants, can similarly recognize social rank of others^7,8^. Studies have shown that normal human subjects exhibit more orientation of visual attention to and better remember faces of higher social rank than those of lower social rank^9–12^. In addition, accumulating evidence from functional imaging studies suggests that social rank recognition is mainly divided into one that is based on social contexts and the other that is based on physical characteristics (physical characteristics provoking social rank, such as difference of body sizes)^5^. Studies have shown that the neural network centered on the medial prefrontal cortex (mPFC) processes social rank recognition with social contexts, whereas the neural network centered on the intraparietal sulcus (iPS), the brain region that mediates mathematical and quantity processing^5^, plays a role in social rank recognition with physical characteristics^5^. In ASD, lower mPFC activation in Theory of Mind tests than typically developing (TD) subjects has been reported^13^. In contrast, ASD subjects demonstrate superior mathematical abilities compared to TD subjects^14^.

Collectively, we hypothesized that imbalance toward lower socially relevant mPFC activity and facilitated mathematically relevant iPS activity, and thereby, disruption of social rank recognition with social contexts, but facilitated social rank recognition with physical characteristics, was observed in ASD. To examine this hypothesis, in this study, we investigated how social rank recognition was altered in school-aged children diagnosed with ASD. Contradicted to the hypothesis, ASD children exhibited similar level of social rank recognition with SC compared to TD children. However, it appeared that the process underlying social rank recognition with social contexts in ASD children was different from that of TD children.

## Results

### Altered social rank recognition in ASD

**Figure 1:**
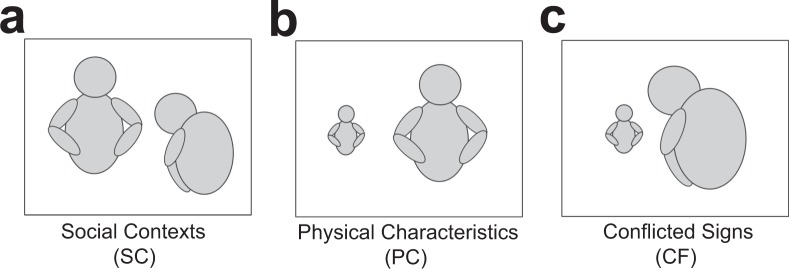
Social rank recognition test. (**a**–**c**) Examples of images with body languages/gestures as social contexts (**a**; SC), physical characteristics (**b**; PC), and conflicts between them (**c**; CF), used in the test.

To investigate social rank recognition in ASD and TD children, we have developed the social rank recognition test, in which social rank recognition was assessed by presenting images illustrating two characters, along with signs of relationships implying that one was dominant and the other was subordinate. These signs were divided into (i) body languages/gestures as social contexts (SC; Fig. 1a), (ii) physical characteristics, such as body sizes (PC; Fig. 1b), and (iii) conflicted social and physical signs (CF; Fig. 1c). Participants rated social rank of two characters in each image, and the difference of rating scores (DRS) between the characters was considered as social rank recognition, with higher DRS indicating more robust social rank recognition.

**Figure 2:**
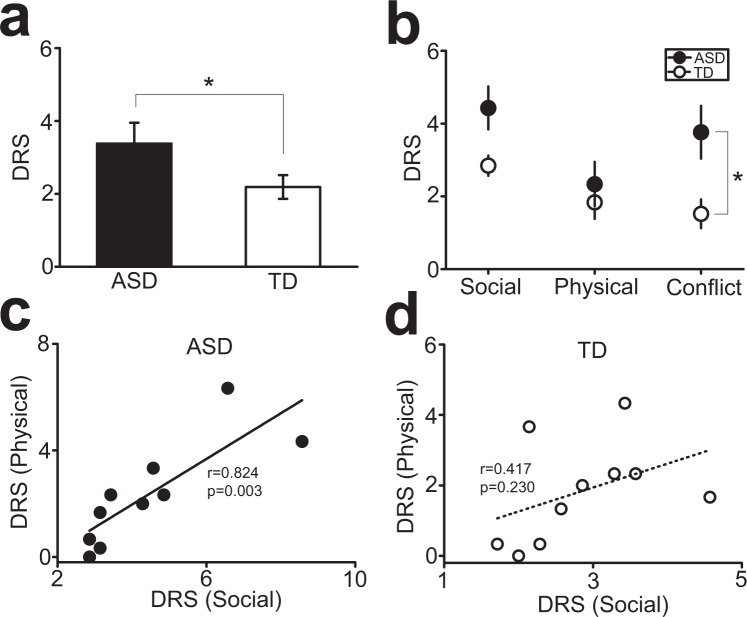
Social rank recognition in ASD and TS children. (**a**) A graph showing difference of rating scores (DRS) for all images with SC, PC, and CF, combined. (**b**) A graph showing DRS separately for SC, PC, and CF. (**c**,**d**) Graphs showing correlations of DRS between for SC and PC in ASD (**c**) and TD (**d**) children, respectively.

DRS for all of SC, PC, and CF conditions combined was significantly higher in ASD than that in TD children (unpaired t-test, t_18_ = 2.20, p = 0.041, *d* = 0.99; Fig. 2a). When DRS was separately analyzed for each of SC, PC, and CF conditions, ASD children exhibited significantly higher DRS than TD children in the for CF condition (two-way ANOVA, F_2,54_ = 4.41, p = 0.017, *η*^2^ = 0.14, for sign types (SC vs. PC vs. CF); F_1,54_ = 11.1, p = 0.002, *η*^2^ = 0.29, for subject types (ASD vs. TD); F_2,54_ = 1.38, p = 0.261, *η*^2^ = 0.05, for interaction; post-hoc Tukey test, p = 0.046 for ASD vs. TD in CF; Fig. 2b). A robust correlation was also observed between DRS for the SC and PC conditions in ASD children (r = 0.824, p = 0.003; Fig. 2c), which remained statistically significant even after two outliers with large DRS compared to others were removed (r = 0.801, p = 0.017). In contrast, such correlation was not observed in TD children (r = 0.417, p = 0.230; Fig. 2d), suggesting a possibility that social rank recognition of ASD children may be different from that of TD children.

### Altered associations between social rank recognition and optical illusion in ASD

To further explore how social rank recognition of ASD and TD children might differ, multiple regression analysis was conducted to evaluate associations between DRS in the social rank recognition test and several factors that might be related to ASD, social rank, or both of them. These factors included Autism Spectrum Quotient (AQ^15,16^), urine serotonin (5-HT) concentration, and personality traits for aggression and sociality. In particular, aggression and sociality have been show to play some roles in social hierarchy of both humans and animals^4,17–19^, whereas the roles of 5-HT are suggested in both ASD^20^ and social hierarchy^21^. In addition, DRS for optical illusion was further examined, and included in this analysis, to evaluate whether social rank recognition, especially with PC, was literally related to perceived size difference of objects.

Significant difference between ASD and TD children was observed in AQ and urine 5-HT concentration, but not other factors (Table 1). However, such difference of urine 5-HT concentration appeared to be primarily due to a subset of ASD children exhibiting very high 5-HT concentration within the group (i.e. hyperserotonemia; Suppl. Fig. S1a,b). Multiple regression analysis revealed significant associations between DRS in the social rank recognition test and that in the optical illusion test (Table 2). In ASD children, associations were observed for both SC and PC conditions, whereas such association was found only for the PC condition in TD children (Table 2), suggesting that social rank recognition with PC is indeed dependent on perceived size difference of objects in both ASD and TD children, whereas, in ASD, consistent with the abnormal correlation between DRS for the SC and PC conditions in ASD, but not TD children, social rank recognition with SC appears to involve the system that processes perceived size difference.

**Table 1:** Comparison of several factors associated with ASD, social rank, or both of them, in ASD and TD children.

	ASD	TD	*t-value*	*p*
	(*mean* ± *s*.*e*.*m*.)			
AQ	26.9 ± 1.70	11.8 ± 2.25	5.39	<0.001*
Optical Illusion	3.06 ± 0.74	2.03 ± 0.31	1.29	0.21
Aggression	3.78 ± 1.06	1.25 ± 0.53	2.05	0.059
Sociality	5.00 ± 0.99	2.75 ± 1.26	1.42	0.18
Urine 5-HT (μg/ml)	0.111 ± 0.029	0.047 ± 0.001	2.16	0.044*

^*^Statistical significance.

**Table 2:** Multiple regression analysis for associations between social rank recognition and several factors of interest.

	ASD				TD			
	Social contexts		Physical characteristics		Social contexts		Physical characteristics	
	*β*	*p*	*β*	*p*	*β*	*p*	*β*	*p*
AQ	−0.35 ± 0.38	0.42	−0.37 ± 0.66	0.62	0.83 ± 1.87	0.70	−0.37 ± 0.13	0.099
Optical Illusion	1.31 ± 0.24	0.012*	1.38 ± 0.41	0.045*	0.26 ± 1.10	0.84	0.75 ± 0.10	0.015*
Aggression	−0.14 ± 0.45	0.78	−0.37 ± 0.78	0.67	0.61 ± 2.12	0.80	−0.089 ± 0.11	0.51
Sociality	−0.39 ± 0.14	0.064	−0.27 ± 0.23	0.33	−0.77 ± 1.48	0.65	0.084 ± 0.11	0.53
Urine 5-HT	0.033 ± 0.21	0.88	−0.088 ± 0.37	0.82	1.02 ± 0.83	0.34	−0.25 ± 0.12	0.16

Mean ± s.e.m.; *Statistical significance.

## Discussion

In this study, we found that, (i) somehow contradicted with the hypothesis, ASD children exhibited more robust social rank recognition than TD children, especially when SC and PC signs conflicted with each other; (ii) a correlation between social rank recognition in the SC and PC conditions was observed in ASD, but not in TD children; and (iii) associations between DRS in social rank recognition with PC and that in the optical illusion test were observed both in ASD and TD children. In contrast, association between DRS in social rank recognition with SC and that in the optical illusion test was also observed in ASD, but not TD, children. The fist finding is consistent with previous studies in healthy human subjects showing that social rank recognition with SC and PC are processed in distinct neural systems. Thus, the current finding suggests that these neural systems may compete with each other in social rank recognition with conflicted SC and PC in TD children. In contrast, such competition of the systems may be absent in ASD. Such difference of social rank recognition between ASD and TD children was further supported by the second finding. The absence of correlation between DRS in the SC and PC conditions in TD children is again consistent with processing of social rank recognition with SC and PC in distinct systems, whereas the abnormal correlation between DRS in the SC and PC conditions in ASD suggests that, in ASD children, social rank recognition with both SC and PC may be processed by the mutual neural system. These first and second findings were further supported by the third finding, unveiling the abnormal association between DRS in the SC condition and optical illusion in ASD, but not TD, children. This suggests that, in ASD children, social rank recognition with SC may be processed in the system that processes perceived size difference of objects, such as the one for social rank recognition with PC. Collectively, ASD children might perceive social rank of others with the signs of SC by developing a compensatory strategy utilizing the system that normally processes social rank recognition with PC, which could be as a consequence of impaired social information processing system.

Accumulating evidence suggests that mPFC and iPS are involved in social rank recognition, but distinctly for processing of that with SC and PC, respectively, in normal subjects^5^. Thus, our study expects to observe that both ASD and TD children may exhibit iPS activation in social rank recognition with PC. However, TD children may exhibit mPFC activation in social rank recognition with SC, whereas ASD children may exhibit iPS activation in social rank recognition with SC, instead of mPFC.

This study is rather at a preliminary stage, given a number of subjects participated in the study is relatively small. Since no clear age and gender difference in social rank recognition was observed among such a small number of participants, gender and age difference was not considered in this study. However, age and gender difference may emerge if a study is conducted with larger number of subjects. Age of participants varied from 7 to 19 years old, with the average ages of 15.4 ± 0.93 and 13.3 ± 0.60 years old in ASD and TD children (t_19_ = 1.83, p = 0.083), respectively. These school-aged children were recruited into this study, since children around at this age become more sentient and are exposed to complex social environments, including existence of hierarchy, in their daily school life. Whether alterations of social rank recognition alterations observed in this study are similar in adult or younger-age ASD subjects remain unknown, and need to be addressed in a future study.

In conclusion, our study suggests that deficits of social information processing could be compensated by other neural systems when it comes to social rank recognition in ASD. Thus, ASD subjects may perceive social rank of other individuals more strictly, but in a different manner from normal subjects, especially when such recognition is based on social signs.

## Methods

### Subjects

This study was conducted in accordance with the Declaration of Helsinki, and all experimental procedures were approved by the Human Research Ethics Committee of Kyoto University Primate Research Institute. Written informed consents, and written informed ascents when applicable, were obtained from participants or/and their parents. Eleven ASD children who were diagnosed with ASD at clinics (8 males, 3 females; from 7 to 19 years old) and 10 TD children (5 males, 5 females; from 11 to 16 years old) were recruited under the Kyoto University Kokoro Research Center Collaborative Research Project. The data from one ASD subject was dropped from further analysis, due to invariable rating of the maximal score for all images in the social rank recognition test.

### Social rank recognition test

We have conceived the social rank recognition test to evaluate recognition of social rank of other individuals. This test consisted of 18 images presented on the computer monitor, with each of image including two persons, puppets, or personificated animals, and positioning one on the left side and the other on the right side of the image. In each image, these two characters exhibit a sign of their relationship implying that one was dominant and the other was subordinate. The signs were divided into 3 categories; (i) body languages/gestures as social contexts, e.g. one character is scolding the other character (SC; Fig. 1a), (ii) physical characteristics, e.g. substantially different body sizes of two characters (PC; Fig. 1b), and (iii) conflicted social and physical signs, e.g. a smaller character is scolding a larger character (CF; Fig. 1c). The test included 7 images with SC, 6 images with PC, and 5 images with CF. Participants were asked to rate rank of two characters on the left and right sides, respectively, in each image, by dividing (to the first decimal place, if necessary) a total of 10 points allotted to each of the left and right characters, such that the difference of rating scores (DRS) given between two characters was considered as social rank recognition, with higher DRS indicating more robust recognition. Although images used in the conflict condition contained both SC and PC signs at the same time, DRS was not scored separately for each of them, but scored as a whole, in this condition.

### Optical illusion test

The optical illusion test was similar to the social rank recognition test, but in this test, 10 optical illusions (Ebbinghaus, Delboeuf, Jastrow, Müller-Lyer, Ponzo, Vertical–horizontal, Oppel–Kundt, Sander, Checker shadow, and Bezold) were presented. Participants were asked to rate perceived difference (in length, size, or color strength) of two objects in each illusion by dividing a total of 10 points allotted for each image, such that the DRS given between two objects was considered as recognition of size difference, with higher DRS indicating more robust recognition.

### Personality questionnaire

Personality traits of participants were evaluated using the paper-based Japanese questionnaire, TS-type Infant and Child Personality Diagnostic Test^22^. This test included 139 questions regarding personality traits of infants and children, which were evaluated by their parents with an answer “yes” or “no”. The test assessed 10 personality traits (self-assertion, nervousness, emotional instability, self-control, dependency, regression, aggression, sociality, adaptation in family environments, and adaptation in school environments), of which aggression and sociality were evaluated in this study. The number of the answer “yes” in each personality trait was calculated as a raw score, with the higher score indicating the more problematic trait, i.e., higher scores in aggression and sociality indicate more aggressive and less social personality, respectively. Although raw scores are normally converted into a percentile rank ranging from 1 to 99% of population level using a conversion table included in the test kit, raw scores were used to evaluate associations with DRS in the social rank recognition test in this study.

### Urine 5-HT assay

5-HT concentrations in urine samples obtained from participants were measured using enzyme-linked immunosorbent assay (ELISA). Urine collection kits were distributed to participants in advance before psychological tests, and urine were sampled by the participants themselves in the morning of the day that psychological tests were conducted. All urine samples were collected within 6 hours, at which 10 ml of urine was mixed with the equal amount of 6 M acetic acid, and stored at −20 °C in the freezer until the day of processing for ELISA. Sample processing was conducted using a commercially available human 5-HT ELISA kit (Catolog #ARG80480; Arigo Biolaboratories, Taiwan, ROC) according to the manual. After processing, ELISA plates were read using iMark microplate Reader (Bio-rad, Hercules, CA).

### Data analysis

Statistical analysis of the data was conducted using unpaired t-test or ANOVA with post-hoc Tukey test. Multiple regression analysis was employed to examine associations between social rank recognition and other factors of interest. Statistical analysis was conducted using the statistical analysis software, Statistica (StatSoft, Tulsa, OK, USA).

## Supplementary information

Supplementary Information

## Data Availability

The datasets generated and analysed during the current study are available from the corresponding author on reasonable request.
