# Supplementary material for: Atypical Social Rank Recognition in Autism Spectrum Disorder
Source: Sci Rep. 2019 Oct 30;9:15657. doi: 10.1038/s41598-019-52211-8 (PMC6821924; doi:10.1038/s41598-019-52211-8)
Supplement: Supplementary file 1 — Supplementary Information [file 41598_2019_52211_MOESM1_ESM.pdf]

Supplemental Information

Atypical Social Rank Recognition in Autism Spectrum Disorder

S. Ogawa, M. Iriguchi, Y-A. Lee, S. Yoshikawa, & Y. Goto

Supplemental Figure S1

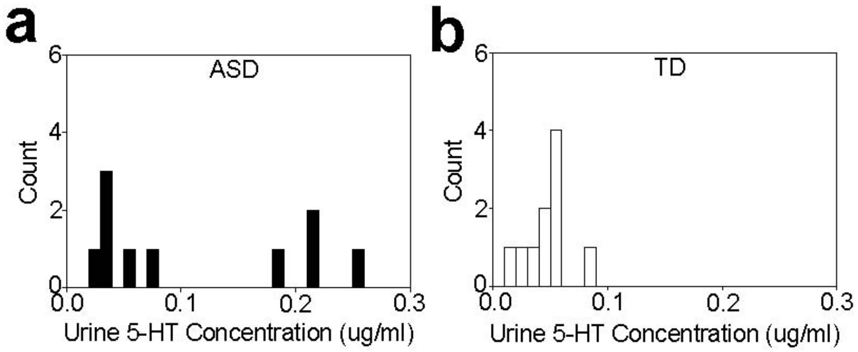

Supplementary Figure S1. Urine serotonin (5-HT) concentration in autism spectrum disorder (ASD) and typically developing (TD) children.
(a, b) Histograms showing distributions of ASD (a) and TD (b) children.
